# Environmental enrichment: a systematic review on the effect of a changing spatial complexity on hippocampal neurogenesis and plasticity in rodents, with considerations for translation to urban and built environments for humans

**DOI:** 10.3389/fnins.2024.1368411

**Published:** 2024-06-11

**Authors:** Mohamed Hesham Khalil

**Affiliations:** Department of Architecture, University of Cambridge, Cambridge, United Kingdom

**Keywords:** spatial complexity, environmental enrichment, housing condition, hippocampus plasticity, neurogenesis, nerve growth factor, anxiety behavior, architectural design

## Abstract

**Introduction:**

Hippocampal neurogenesis is critical for improving learning, memory, and spatial navigation. Inhabiting and navigating spatial complexity is key to stimulating adult hippocampal neurogenesis (AHN) in rodents because they share similar hippocampal neuroplasticity characteristics with humans. AHN in humans has recently been found to persist until the tenth decade of life, but it declines with aging and is influenced by environmental enrichment. This systematic review investigated the impact of spatial complexity on neurogenesis and hippocampal plasticity in rodents, and discussed the translatability of these findings to human interventions.

**Methods:**

Comprehensive searches were conducted on three databases in English: PubMed, Web of Science, and Scopus. All literature published until December 2023 was screened and assessed for eligibility. A total of 32 studies with original data were included, and the process is reported in accordance with the PRISMA (Preferred Reporting Items for Systematic Reviews and Meta-Analyses) statement and checklist.

**Results:**

The studies evaluated various models of spatial complexity in rodents, including environmental enrichment, changes to in-cage elements, complex layouts, and navigational mazes featuring novelty and intermittent complexity. A regression equation was formulated to synthesize key factors influencing neurogenesis, such as duration, physical activity, frequency of changes, diversity of complexity, age, living space size, and temperature.

**Conclusion:**

Findings underscore the cognitive benefits of spatial complexity interventions and inform future translational research from rodents to humans. Home-cage enrichment and models like the Hamlet complex maze and the Marlau cage offer insight into how architectural design and urban navigational complexity can impact neurogenesis in humans. In-space changing complexity, with and without physical activity, is effective for stimulating neurogenesis. While evidence on intermittent spatial complexity in humans is limited, data from the COVID-19 pandemic lockdowns provide preliminary evidence. Existing equations relating rodent and human ages may allow for the translation of enrichment protocol durations from rodents to humans.

## 1 Introduction

Neurogenesis is the process by which new neurons are formed in the brain, in particular in the hippocampus and the olfactory bulb (Kempermann, 2022a). The hippocampus is involved in learning, memory, and spatial navigation. Neurogenesis in this region does not cease after development, but is rather modulated by environmental factors across the whole lifespan. Therefore, stimulating the brain to generate new neurons in the hippocampus through spatial experiences is a critical step for humans at all life stages.

Adult hippocampal neurogenesis (AHN) is the mammalian brain’s ability to generate neurons in the dentate gyrus of the adult hippocampus throughout life and beyond the early developmental phases (Kempermann, 2002; Christian et al., 2014). Evidence suggests that newborn neurons might play crucial roles in certain functions of the hippocampus, such as pattern separation, i.e., the ability to distinguish and store similar but not identical inputs of sensory information into distinct representations. Moreover, newborn neurons at various maturation states can exhibit distinct contributions to learning and memory (Deng et al., 2010). A recent study by Kempermann (2022b) has shown that neurogenesis promotes the flexible integration of novel information into familiar contexts and improves episodic memory, a type of memory which relates to autobiographical information (d’Isa et al., 2011).

For decades, researchers have been able to study neurogenesis in non-human subjects, specifically rodents (Toda et al., 2019). Recently, evidence for adult hippocampal neurogenesis (AHN) in humans suggests that it persists until the tenth decade of human life, and impairments in this process are associated with memory encoding dysfunction, mood disorders, and dementia (Mu and Gage, 2011; Babcock et al., 2021; Moreno-Jiménez et al., 2021). An increase in AHN is assumed to be positively correlated with reduced depressive and anxiety behaviors (Snyder et al., 2011; Hill et al., 2015), but direct evidence for this role is lacking. For instance, one of the roles of antidepressants is to increase neurogenesis in order to contribute to improving cognition and mood (Tartt et al., 2022). Beyond antidepressants, mood stabilizers also can boost neurogenesis with varying results (Carli et al., 2021).

Regarding the role of neurogenesis in integrating elements from both the internal and external environment to adjust brain functions, a recent review suggests that neurogenesis could be the ultimate cellular process (Alonso et al., 2024), raising questions about the influence of the external environment on neurogenesis, both positively and negatively, to prevent and treat depressive and anxiety symptoms. In mice, a growing body of knowledge shows the impact of different environmental enrichment (EE) interventions, including running wheels, in stimulating neurogenesis, which has been found to enhance learning (Olson et al., 2006; Fabel et al., 2009; Garthe et al., 2016; Grońska-Pęski et al., 2021). However, many studies confound structural EE with opportunities for physical activity, making it challenging to understand the effects of one factor or the other on neurogenesis. In humans, there are almost no studies available due to the difficulty of studying humans in controlled environments, and recent rodent studies highlight issues of translatability (Kempermann, 2019; Rojas-Carvajal et al., 2022). Although there is a significant gap in the existing literature between rodents and humans, Clemenson et al. (2015) have explored the potential impact of EE without running wheels on neurogenesis, drawing comparisons between evidence from mice and indirect findings from studies on humans, suggesting that neurogenesis, pattern separation ability, and other hippocampal plasticity outcomes are more similar than different between mice and humans.

Some of the problems that have widened the gap between humans and rodents include claims that humans live in already enriched environments, the impossibility of controlling humans in laboratory environments like rodents, and the difficulty of observing outcome changes in humans.

Firstly, we debunk the notion that humans live in environments that could not be further enriched. Early rodent research on EE focused on comparing the effects of EE to standard housing, showing positive correlations between the former condition and neurogenesis (Kempermann et al., 1997). More recent studies have begun to explore the nuanced effects of novelty and complexity compared to standard housing (Bohn et al., 2023), revealing that mice indeed prefer structurally enriched cages. However, it was found that in the third week, mice spent more time in the extra cage which was connected to their home-cage if this extra cage featured a changing complexity rather than a constant complexity. As humans often reside in the same house for years, sometimes decades, it appears that there would certainly be a margin for enrichment, especially when the latest literature on mice reveals nuanced differences between fixed complex and novel complex environments.

The second argument posits that it is impossible to control humans in their environments like rodents, a notion we support but also aim to debunk at the same time. In certain situations, for instance during the COVID-19 lockdown, the human home mostly mirrored the conditions of a rodent home-cage, with numerous studies reporting mental distress and negative psychological symptoms associated with lockdown at home (Novotný et al., 2020; Ozamiz-Etxebarria et al., 2020). Increased anxiety and depressive symptoms have been reported during this period (Benke et al., 2020; Every-Palmer et al., 2020; Di Blasi et al., 2021). The role of the human home in stimulating neurogenesis, however, remains ambiguous and unknown, though it is likely that the complexity of the home environment, even if initially classified as complex, tends to remain relatively fixed. A single room or even a city represent further scales of complexity as if humans lived in multiple cages juxtaposed with each other.

Given that humans spend a portion of their time outside their homes and workplaces, it is plausible to consider isolating the city as a macrocosm of a more complex cage. Shin et al. (2024) aimed to understand the impact of environmental factors on Alzheimer’s disease (AD), specifically focusing on spatial complexity as an important but understudied factor in the routine navigation of human environments. They quantified local geospatial properties (road network and landmark elements) to define environmental complexity, based on an earlier study allowing for the calculation of a geospatial environmental complexity index (Yuan and Kennedy, 2023). A sample of older adults was recruited, and it was found that greater environmental complexity was associated with fewer AD diagnoses and better spatial behavioral performance. Furthermore, several studies have shown that London taxi drivers, who extensively navigate London, exhibit changes in their hippocampi (Woollett and Maguire, 2011). Conversely, stress leads to loss of adult hippocampal neurogenesis and consequent reduction of hippocampal volume (Schoenfeld et al., 2017). However, in these studies complexity is not explicitly defined in terms of change, as sought in the present study.

Now that we have debunked the notion that humans live in non-enrichable environments and addressed the difficulty of controlling human subjects, it remains important to highlight the feasibility of testing outcomes in humans under these circumstances. Longitudinal studies have shown that magnetic resonance imaging (MRI) can be employed to illustrate differences hypothesized to be correlated with the quantified independent variable, which we introduce in the present study as the change in spatial complexity. However, the expanding research on human subjects has facilitated the tracking of adult hippocampal neurogenesis (AHN) also through more sophisticated methods, such as the molecular profiling of specific cell types in postmortem brain samples (Terreros-Roncal et al., 2023; Tosoni et al., 2023).

On one hand, several studies on rodents have demonstrated that exercise-mediated neurogenesis is associated with an increase in brain-derived neurotrophic factor (BDNF) levels (Liu and Nusslock, 2018), while a systematic review by Barros et al. (2019) found few studies correlating the cellular mechanisms involved in hippocampal neurogenesis with BDNF expression when rodents are exposed to EE. We already have evidence that physical activity, not necessarily structured exercise, has positive effects on increasing BDNF levels in animals and humans (Zoladz and Pilc, 2010), including healthy humans (Huang et al., 2014). For instance, a study by Park et al. (2020) involved twice-weekly gardening activity sessions (lasting an average of 60 min per session) for elderly humans, which correlated with an increase in BDNF levels. Similarly, also the effects of EE itself could be experimented in humans employing BDNF levels as outcome.

On the other hand, there are already validated psychometric tools that can be utilized for evaluating the effects of EE in daily human life, such as the Florida Cognitive Activities Scale (FCAS), the Multidimensional Social Integration in Later Life Scale (SILLS), and the International Physical Activity Questionnaire (IPAQ) (Flores-Ramos et al., 2022), along with the Generalized Anxiety Disorder 7 (GAD-7) inventory (De Hoyos et al., 2023). Notably, these psychometric studies have measured cognitive, social, and physical activities, but not the spatial complexity of life environments (Flores-Ramos et al., 2022), further underscoring the gap in the literature. Other studies have concentrated on social support, novelty, and open spaces as correlates of anxiety in patients with endometriosis and other inflammatory, painful disorders (De Hoyos et al., 2023; Nieves-Vázquez et al., 2023), validating the use of GAD-7 while focusing on a different scope of EE. The National Center for Health Statistics (NCHS) in the United States employed a shorter version of the GAD-7, the GAD-2, along with the short form of the Patient Health Questionnaire (PHQ-2), to explore depression symptoms during the COVID-19 lockdown. The longer form PHQ-8 was instead used by the Office for National Statistics in the United Kingdom.

The present paper systematically reviews the existing body of knowledge on evidence-based research regarding the effects of spatial complexity on neurogenesis. Moreover, we analyze the concept of spatial complexity and we try to define the factors that may modulate its pro-neurogenic effect. Additionally, we propose a regression model that may be helpful both to describe rodent paradigms of EE and to translate findings from rodents to humans. Finally, we provide suggestions for future research on human subjects seeking to investigate the impact of changing physical or structural conditions in human housing environments and their potential to improve neurogenesis and associated plasticity outcomes. Indeed, while in rodents EE studies have been originally conducted more than 60 years ago, highlighting the positive effects of environmental complexity on brain chemistry (Krech et al., 1960), EE in humans is still poorly investigated.

## 2 Method

### 2.1 Research strategy

The search query was conducted across three databases: PubMed, Web of Science, and Scopus. The data is reported in accordance with the PRISMA (Preferred Reporting Items for Systematic Reviews and Meta-Analyses) statement and checklist (Moher, 2009). The search included all articles found and published up to December 2023, using the following keywords: (house OR housing OR “housing w/ space” OR “spatial complexity” OR “architectural complexity” OR “environmental complexity” OR “physical enrichment” OR “structural enrichment” OR “spatial enrichment”) AND (neurogenesis OR “neurogenesis w/ BDNF” OR “hippocampus w/ neurogenesis” OR “hippocampal w/ neurogenesis” OR “hippocampus w/ plasticity” OR “hippocampal w/ plasticity” OR “spatial pattern separation”). While research on neuroplasticity is relatively recent, there are no restrictions on the publication year.

### 2.2 Research framework

The Population, Intervention, Comparison, Outcome (PICO) framework was adopted to capture each key element required for the research aim (Schardt et al., 2007; Farrugia et al., 2010). Firstly, Population (P): Adult and aged rodents with healthy brains (non-diseased or diagnosed with a disorder, pathology, seizure or epilepsy), without the influence of other substances (alcohol, cocaine, etc.), not diagnosed with diabetes or cardiovascular disease, and not pregnant or in maternity in the case of female rats. Secondly, Intervention (I): Either short- or long-term EE intervention focusing on spatial or structural complexity of a housing space without challenging subjects with stressors, serotonin depletion, etc. Thirdly, Comparison (C): Standard housing or other EE conditions. Lastly, Outcome (O): Neurogenesis and associated hippocampal plasticity outcomes, if any.

### 2.3 Screening and inclusion criteria

The screening criteria considered only English peer-reviewed journal articles eligible, while gray literature references (books, book chapters, conference papers, notes, retracted papers, and reviews) were excluded from consideration. All fields of study were considered, and no area of study was excluded. After including articles that met the inclusion and exclusion criteria, duplicated articles across the three databases were removed before proceeding with data extraction and selection of articles for full-text reading.

### 2.4 Eligibility and article selection

Articles that passed the preliminary inclusion and exclusion screening criteria were selected for further evaluation in two phases. Initially, articles were assessed based on title, abstract, and keywords to ensure alignment with the research aim for which the search strategy was designed. Subsequently, selected articles for full-text reading underwent additional scrutiny for eligibility and were subjected to quality assessment. Following this, the eligible full-text studies underwent a thorough examination of their reference lists, citations, and relevant articles on the respective database pages. This approach was employed as a snowball method to identify additional relevant articles that still met the inclusion criteria. Data were then extracted and assessed for eligibility using the same criteria.

### 2.5 Study quality assessment

The quality of the selected articles for this systematic review was evaluated by considering components that may affect the internal validity of experimental animal studies. This was achieved by assessing the included studies according to the recommendations of the SYRCLE risk of bias tool (Hooijmans et al., 2014). Among several items, the included studies were evaluated based on baseline characteristics, randomization, blinding, temperature, lighting, housing conditions, nutrition, attrition, reporting, and committee on institutional ethics.

## 3 Results

**Figure 1:**
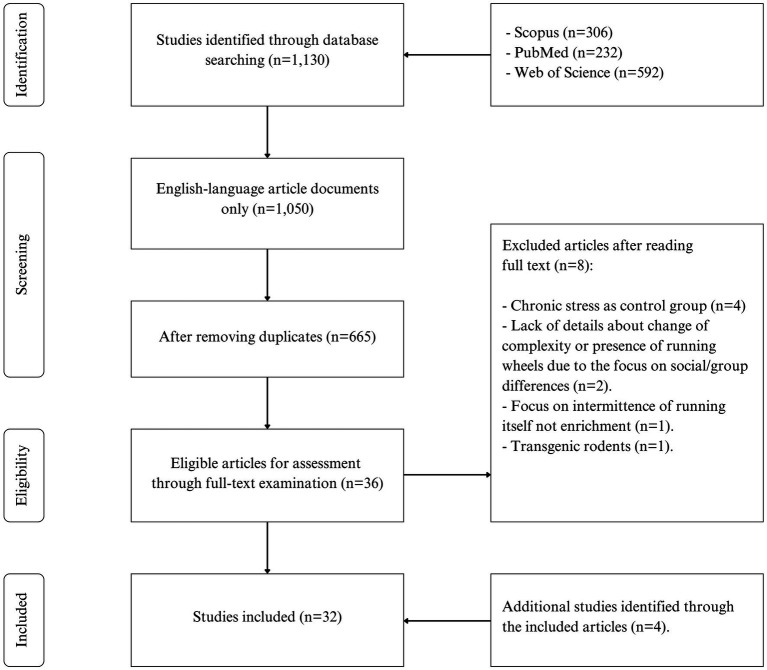
Flow diagram of the study selection process.

A total of 1,130 studies were obtained from three databases (Scopus, PubMed, Web of Science), which were reduced to 665 after limiting results to English-language journal articles and removing duplicates. Following title and abstract screening, 36 articles were included for full-text reading, but some did not meet the eligibility criteria (8 articles). With an additional 4 studies handpicked and assessed for eligibility through snowball acquisition, the final number of included articles increased to 32. The PRISMA flow chart of study selection is presented in Figure 1.

Overall, a sufficient number of studies (*n* = 32) were found to support the argument that changing spatial complexity stimulates neurogenesis by elucidating nuanced intervention differences and revealing multiple associated hippocampal plasticity outcomes. Table 1 lists all included studies, presenting the rodents’ gender, strain, study duration, complexity intervention, neurological outcomes, and behavioral outcomes if present. Table 2 presents the results of the risk of bias assessment using SYRCLE for the included studies.

**Table 1:** List of included studies.

Source	Species	Age†	Duration*	Spatial complexity conditions	Neurobiological results	Behavioral results
Kempermann et al. (1997)	Female C57BL/6 (Sprague Dawley) mice	3 weeks old	40 days	Twelve mice were divided into standard cages housing four mice each, while another twelve were housed together in a specially designed cage measuring 1m^2^. This special cage featured paper tubes, nesting material, rearrangeable plastic tubes, a tunnel with multiple openings, and a running wheel.	Significantly more neurons found in the dentate gyrus of mice exposed to an enriched environment (EE) compared with littermates housed in standard cages. The former group had a larger hippocampal granule cell layer and 15% more granule cell neurons in the dentate gyrus.	—
Kempermann et al. (1998a)	C57BL/6 mice	6 and 18 months old	40 or 68 days	Twelve mice per age group were housed in four standard cages, while 13 per age group were housed in an EE, which included objects as toys, a rearrangeable set of tunnels and a running wheel.	Neurogenesis decreased with increasing age, but with EE, relatively more cells differentiated into neurons, resulting in a threefold net increase of neurons in aged mice (105 vs. 32 cells) and a more than twofold increase in adult mice (648 vs. 285 cells). Stimulation of adult and aged mice by switching from standard housing to an EE for 68 days resulted in an increased survival of labeled cells.	—
Kempermann et al. (1998b)	129Sv/J mice	3 weeks old	40 days	Thirteen mice lived in an EE, composed of one large cage with rearrangeable set of tunnels, toys and running wheels, while 12 control mice were held 3 per cage in standard lab cages.	EE 129/SvJ mice had twice as many proliferating cells in the dentate gyrus compared to mice in standard housing. EE stimulation resulted in 67% more new neurons.	Spatial memory and motor learning were improved.
Nilsson et al. (1999)	Female Sprague–Dawley rats	3 months old	4–8 weeks	Ten rats were placed in smaller cages compared to 12 rats in larger cages with ladders, several platforms, nesting material and objects with new toys introduced every 3–4 days.	Environmental cues can enhance neurogenesis in the adult hippocampal region.	Improved spatial memory.
Åberg et al. (2005)	Female Balb/c mice	5 weeks old	8 weeks	Mice were housed in standard conditions or enhanced conditions each as groups of four per cage. After 2 weeks, mice were further assigned to one of six experimental groups till week 8: standard-control, enhanced-control, standard alternated between group and individual housing, enhanced alternated between group and individual housing, and standard alternated between group housing and individual housing with running wheels.	Intermittent individual housing (on every second day) without, but not with, running wheels, increased survival of proliferated cells in the dentate gyrus compared with continuous group housing in either standard or enhanced conditions.	—
Segovia et al. (2006)	Male Wistar rats	2 and 25 months old	8 weeks	Rats were housed as 8 per large cages with running wheels, plastic tunnels, elevated platform and toys changed every 3–4 days (enriched), while the control group were in mesh cages. Each condition was duplicated to house the two age groups.	Aged rats showed decrease of BrdU positive cells in the dentate gyrus, but neurogenesis in the dentate gyrus of both young and old rats was increased in the EE condition. Neurotransmitters glutamate and GABA in the CA3 area of aged rats increased, compensating for the deterioration due to aging.	EE did not increase the learning abilities of aged rats, contrarily to other existing evidence, suggesting that there are other age-related factors.
Leal-Galicia et al. (2007)	Male Wistar rats	1 month old	15 days	Twenty (5 per cage) rats were housed in a large EE cage with elements such as plastic tunnels, balls, bells and running wheels. Elements were placed in different positions every day. Ten rats were kept in conventional conditions as the control group.	New neurons were visualized, with a positive correlation between activity displayed in EE and neurogenesis.	Differences were observed between the activity levels displayed by rats within the enriched housing versus conventional housing, found to be correlated with differences in hippocampal cell proliferation. Exposure to EE reduced anxiety-like behaviors and these reductions were positively linked with hippocampal neurogenesis and testosterone level.
Hattori et al. (2007)	Female C57BL/6 J mice	10 weeks old	5 weeks	The control group housed 3 mice in a housing cage of common size, while the EE group housed 3 mice in a large rodent cage with 3 toys, a running wheel, plastic tube and wooden swing along with wooden products, which were changed once per week.	Increased survival of newborn cells was observed in EE mice, revealing later that the majority of surviving cells were neurons.	EE showed beneficial effects on behavioral despair and habituation to a novel environment.
Leal-Galicia et al. (2008)	Male Wistar rats	3 months old	18 months	The EE consisted of different elements such as plastic tunnels, balls, bells and a running wheel. 20 mice were placed in groups of 5 in the EE system at the hours of highest activity for 2 h daily for 15 consecutive days. To avoid behavioral habituation, rats were intermittently exposed to EE for 3 h once per week during 18 months. In each EE session, objects were changed around to offer a novel environment.	Intermittent lifelong exposure to EE since youth increased the number of new generated neurons within the hippocampus during aging as well as the maintenance of synapses.	Intermittent lifelong exposure to EE since youth had a positive impact on short-memory preservation.
Freret et al. (2012)	Female NMRI mice	3 and 14 month old	6 months	Three groups of mice were used: adult mice in standard enrichment cage, aged mice in standard enrichment cage, and aged mice in EE cage. In the latter 2 groups, mice were middle-aged when allocated to their groups but they were aged considering the strain of mice. Standard conditions included 7–8 mice in a standard size cage, while enriched cages were larger, with 2 floors equipped with objects of different sizes and materials, ladders, ropes, chains, swings, blocks and tubes of different diameters, nesting materials and a large running wheel. The positions of objects, food and water bottles were changed twice a week.	EE attenuated the age-related impairment of neurotransmission in CA1 hippocampal slices, and reversed the decrease in receptor NMDA-R dependent synaptic potentials, but did not prevent long-term potentiation alterations. This study suggests that EE needs to be initiated before the age corresponding to the median lifespan to have an effect on cognitive aging.	EE prevented memory deficits and reduced anxiety as the animal aged.
Birch et al. (2013)	Male Wistar rats	3 months old	2, 3 and 6 weeks	To assess the minimum duration of EE to elicit a cognitive improvement, rats were house in a group of 12 in a standard condition, in a group of 6 in an EE condition for 2 weeks, in a group of 8 in an EE condition for 3 weeks, or in a group of 6 in an EE condition for 6 weeks. EE consisted of various toys, boxes, extra bedding but no running wheels. Toys were chosen to elicit the maximum sensory stimulation and each week these toys were removed, washed and interchanged between cages to ensure consistency in object exposure between groups, while maintaining novelty. Nest boxes were also returned to cages in novel positions.	Increase in nerve growth factor (NGF) concentration, synaptogenesis and subgranular progenitor cell survival in the dentate gyrus of rats enriched. These three effects were all independent from the presence of physical activity.	Rats housed in an EE for 3 and 6 weeks displayed improved recognition memory, while rats in the latter group also displayed improved spatial and working memory.
Fares et al. (2013)	Male Sprague-Dawley rats	3 weeks old	Up to 13 weeks	The Marlau cage, which offers: (1) minimally stressful social interactions; (2) increased voluntary exercise; (3) multiple entertaining activities; (4) cognitive stimulation (maze exploration), and (5) novelty (maze configuration changed three times a week).	Rats housed in Marlau cages exhibited increased cortical thickness, hippocampal neurogenesis and hippocampal levels of transcripts encoding various genes involved in tissue plasticity and remodeling.	Rats housed in Marlau cages exhibited better performances in spatial learning and memory, decreased anxiety-associated behaviors, and better recovery of basal plasma corticosterone level after acute restraint stress.
Speisman et al. (2013)	Male F344 rats	5–8 months and 20–22 months old	10 weeks	Rats were housed either individually or pair housed with 2–3 h of daily access to an EE to provide opportunities for the EE group to engage in a variety of hippocampus-dependent behaviors while limiting them for the individually housed group. The EE environment had a large wooden box, empty water maze tank, or large cage with toys. The environment and toys were randomly rotated daily to maintain novelty.	Neurogenesis decreased with age but enrichment enhanced new cell survival, regardless of age.	Although young rats outperformed aged rats, aged enriched rats outperformed aged individually housed rats on all behavioral measures.
Grégoire et al. (2014)	Male CD1 and C57BL/6 mice	2 months old	4 weeks	Four types of housing environments of similar cage size were used in an “Alternating EE” paradigm: empty environment, locked disc environment, running disc environment, and complex environment. The latter contained an igloo, locked running disc, colored tunnels, green trail, yellow curve, blue u-turn, transparent tee and blue elbow. Tunnels were re-oriented and their conformations re-arranged at each cage alternation (4 times / week).	Running significantly stimulated adult hippocampal neurogenesis, while the environmental complexity, including social interactions and conformational novelty, did not exert a substantial influence on the neurogenic process.	Continuous voluntary running did not yield greater neurogenic effects than intermittent running.
Ramírez-Rodríguez et al. (2014)	Female Balb/c mice	6 months old	35 days	Five mice were housed in a normal cage, while for the EE, 10 mice were housed in a big box with tunnels of different colors, shapes, a running wheel, wooden objects, nesting materials and small plastic houses with stairs. The complexity and distribution of the other materials were changed every third day to avoid habituation.	EE has prevented neuroplastic decline by increasing the formation of both dendritic spines and new neurons in the hippocampus during middle age. Exposure to EE increased BDNF levels.	—
Leger et al. (2015)	Male NMRI mice	10 weeks old	24 h, 1, 3 or 5 weeks	Mice were housed in groups of 12 either in standard condition cages or relatively larger EE cages with various objects of different shapes, sizes, colors, textures, and materials and a large running wheel. Most of the objects and their locations were renewed twice a week to ensure novelty. Not all objects were changed, in order to limit male agonistic behaviors.	The minimal duration of EE to induce neurogenesis in mice was 3 weeks, but the neuronal survival was prolonged only after 5 weeks of EE.	Short enrichment exposure (24 h) was sufficient to improve object recognition memory performance, but a 3-week exposure was required to improve aversive stimulus-based memory performances and to reduce anxiety-like behavior. Behavioral changes after 3-week exposure were supported by higher serotonin levels in the frontal cortex, but independently of neurogenesis.
Hosseiny et al. (2015)	Female C57BL6/J mice	4 or 8 weeks old	4, 6 and 8 weeks	Twelve mice were housed in large-sized cages with nesting materials, running wheels, scales, hammocks, plastic toys and tunnels. Objects were changed twice a week. Control mice were housed in medium-sized cages with 5 mice per cage without objects.	EE enhanced neurogenesis in juvenile but not young adult mice; EE increased the number of synaptic contacts at every stage; long-term potentiation was affected differently by EE depending on its onset and duration.	—
Hüttenrauch et al. (2016)	Female C57BL6/J mice	1 month old	11 months	Mice were randomly distributed between standard housing in a group of 10 mice or enriched housing in a group of 7 and relatively larger cage size. EE cages had three running wheels, nesting material, tunnels, shelters, houses and toys that were changed and rearranged weekly to increase novelty.	The number of CA1 pyramidal neurons remained unchanged in both groups, but the number of dentate gyrus neurons as well as CA1 and DG volumes were significantly increased in the EE group.	EE reduced anxiety and improved spatial reference memory.
Neidl et al. (2016)	Evans rats	18 months old	6 months	Enriched rats were gathered in groups of 10–12 in two larger cages compared to the standard condition cages. EE cages had various objects but no running wheel, and those objects were changed five times a week.	Increased expression of BDNF, demonstrating that aged rats respond to EE by increasing hippocampal plasticity.	EE improved spatial learning and increased acetylation-related events in aged rats.
Zhang et al. (2016)	Male Wistar rats	Adult	1 month	Rats were grouped in a standard environment or EE. The latter was larger in size, with houses, mazes, wheels, chains, sinks, swings, ladders and balls. Toys were changed once or twice per week.	EE increased proliferation, differentiation and survival of newly-formed neurons in the dentate gyrus. EE enhanced protein expression levels of BDNF in the hippocampus.	EE improved spatial memory.
Crouzier et al. (2018)	C57BL/6 J and Swiss OF-1 mice	7–9 weeks old	2 weeks	Mice received training in groups in the Hamlet complex maze: a structure resembling a small village with a central agora, streets expanding from it toward five functionalized houses each with a distinctive use: drinking, eating, hiding, running, and interacting with a stranger.	Training increased hippocampal neurogenesis (cell proliferation and neuronal maturation).	Differences in exploration patterns were observed between C57BL/6 J and Swiss OF-1 strains, but not between males and females.
Birch and Kelly (2019)	Male Wistar rats	Cohort	20 months	Rats were housed in standard housing conditions or EE conditions consisting of various different toys, next boxes, extra bedding and no running wheels or climbing equipment. Toys were chosen to elicit varied sensory stimulation and each week these toys were removed, cleaned and swapped between cages to ensure consistency in object exposure while maintaining novelty. Nest boxes were cleaned and returned in a novel position.	MRI scans were performed at young, middle aged and aged timepoints. EE prevented reduction in NGF in hippocampus, and cell proliferation in the dentate gyrus with aging. The results demonstrate that sensory enrichment alone can have significant benefits.	Age-related decline in recognition, spatial and working memory was prevented by EE.
Sadegzadeh et al. (2020)	Wistar rats	3 weeks old	40 days	Male control rats were kept in standard cages, while female control rats were kept in big cages for synchronization of estrous cycles, and two male and female groups were exposed to EE, which was larger and included various objects (climbing ladders, running wheel, nest box, plastic tubes and balls, and various types of decoration) to motivate exploration. The position of these objects were exchanged once a week.	Housing in the EE during adolescence led to increased BDNF levels in the prefrontal cortex of female rats.	Housing in the EE during adolescence improved passive avoidance memory and increased nociceptive response against thermal stimulus in both sexes.
Sakhaie et al. (2020)	Wistar rats	3 weeks old	40 days	As above	Housing in the EE during adolescence led to increased BDNF levels in the hippocampus of both sexes.	Housing in the EE during adolescence augmented object recognition memory in male rats and reduced anxiety-related behaviors in both male and female rats.
Santoso et al. (2020)	Male Wistar rats	7 months old	8 weeks	The Marlau cage.	The combination group (EE and aerobic exercise) had the highest levels of hippocampal BDNF and NGF.	The combination group (EE and aerobic exercise) had the highest performance in the Y-maze spatial memory test.
Wang et al. (2020)	Male C57BL/6 J mice	4 weeks old	8 weeks	Rats were equally divided into 3 groups: control, EE and social isolation (SI). Mice in the EE group were raised in a large cage and multilayer space with various toys such as houses, running wheels, hammocks, scales, bells, ladders and tunnels. Objects were changed twice a week. Control group rats were in a standard size cage with no objects.	Synaptic plasticity, neurogenesis, and the pattern of neuronal activities were significantly impacted by the housing environment through changing the balance of excitatory and inhibitory synaptic density.	EE improved spatial learning and object recognition memory.
Crouzier et al. (2020)	Male C57BL/6 J mice from different countries	7–9 weeks old	2 weeks	Male mice were trained in groups in the Hamlet maze 4 h/day.	EE induced a strong plasticity, increased BDNF levels in the hippocampus, and increased neurogenesis (proliferation and maturation).	EE augmented resilience to behavioral despair in the forced swim test and to the amnesic effect of scopolamine in the spontaneous alternation Y-maze test.
Oatess et al. (2021)	C57BL/6 J mice	4 weeks old	12 weeks	Mice were assigned to one of two groups: standard housing control group or an enriched group. Both groups were similarly housed in the same cages with the same items yet the EE group received an additional one red acrylic tunnel. Mice were housed in same-sex groups of 3 to mitigate potential male aggression. Cage change took place every 2 weeks with tunnels replaced at each change.	Simple structural enrichment had no effect on hippocampal neurogenesis and on physiologic markers of stress (adrenal gland weight, plasma corticosterone concentration, and neutrophil:lymphocyte ratio).	Simple structural enrichment reduced anxiety in the open field test.
Resasco et al. (2021)	Swiss mice	3 to 4 weeks old	57 days	Female mice were housed in standard-sized cages with no enrichment, simple enrichment or complex enrichment.	Exposure to both simple and complex EE conditions resulted in a significant enhancement of neurogenesis, emphasizing the potential of minor changes in the environment to influence neural processes and promote hippocampal health.	Mice subjected to complex EE exhibited reduced anxiety-like behavior.
Cabrera-Muñoz et al. (2022)	FVB/N mice	23 days	30 days	Mice were grouped in male standard housing, female standard housing, male EE, and female EE. The EE environment contained large boxes with running wheels and tunnels of different colors and shapes. The complexity of the tunnels was modified every third day. Standard housing was a cage of size similar to the EE cage, but with no enrichment objects.	EE increased neurogenesis in both sexes. Neural circuits showed lower activation in the amygdala of enriched males and higher activation in enriched females than respective controls. Enriched females showed higher activation in the frontal cortex without difference in males, while insular cortex was less activated in females than males.	EE increased social interaction in females, but increased agonistic-like behavior in males, as assessed through the resident-intruder test for social behavior.
Ramírez-Rodríguez et al. (2022)	Male Balb/c mice	10 weeks old	42 days	Mice were housed in one of 5 groups: standard conditions without EE, constant EE complexity, gradual increase of EE complexity followed by gradual decrease, graduate increase of EE complexity followed by constant EE, and constant EE followed by a gradual decrease.	Voluntary physical activity alone or in the context of a complex environment increased doublecortin cell maturation in the granular cell layer of the hippocampus.	Any change in environmental complexity over time reduced anxiety.
Funabashi et al. (2023)	Male C57BL/6 J mice	11 weeks old	36 days	The housing environment of the EE mice was gradually enriched by enlarging the housing space and adding objects: standard cage (5 days), large cage (5 days), extra-large cage (5 days) and extra-large cage + objects (21 days).	EE enhanced hippocampal neurogenesis.	Enlarged housing spaces and the placement of a variety of objects did not increase physical activity in mice.

†At the time of the start of the spatial complexity intervention.

*Duration of exposure to the spatial complexity intervention.

**Table 2:** Risk of bias assessment using the SYRCLE tool. (+) = described; (−) = not described; (?) described, but the method was not reported.

List of authors	Baseline	Randomization	Blinding	Temperature	Lighting	Housing condition	Nutrition	Attrition	Reporting	Ethics
Kempermann et al. (1997)	+	?	−	−	−	+	+	+	+	−
Kempermann et al. (1998a)	+	−	−	−	−	+	+	+	+	−
Kempermann et al. (1998b)	+	?	−	−	−	+	+	+	+	−
Nilsson et al. (1999)	+	?	−	−	−	+	+	+	+	+
Åberg et al. (2005)	+	−	−	−	+	+	+	+	+	+
Segovia et al. (2006)	+	−	−	+	+	+	+	+	+	+
Leal-Galicia et al. (2007)	+	−	−	−	+	+	+	+	+	+
Hattori et al. (2007)	+	−	−	+	+	+	+	+	+	+
Leal-Galicia et al. (2008)	+	−	−	−	+	+	+	+	+	+
Freret et al. (2012)	+	−	+	+	+	+	+	+	+	+
Birch et al. (2013)	+	−	−	+	+	+	+	+	+	+
Fares et al. (2013)	+	?	+	+	+	+	+	+	+	+
Speisman et al. (2013)	+	−	−	−	+	+	+	+	+	+
Grégoire et al. (2014)	+	?	+	−	−	+	+	+	+	+
Ramírez-Rodríguez et al. (2014)	+	−	−	+	+	+	+	+	+	+
Leger et al. (2015)	+	−	+	+	+	+	+	+	+	+
Hosseiny et al. (2015)	+	−	+	+	+	+	+	+	+	+
Hüttenrauch et al. (2016)	+	?	−	−	−	+	+	+	+	+
Neidl et al. (2016)	+	?	+	+	+	+	+	+	+	+
Zhang et al. (2016)	+	?	+	+	+	+	+	+	+	+
Crouzier et al. (2018)	+	−	+	+	+	+	+	+	+	+
Birch and Kelly (2019)	+	−	−	+	+	+	+	+	+	+
Sadegzadeh et al. (2020)	+	?	−	+	+	+	+	+	+	+
Sakhaie et al. (2020)	+	−	−	+	+	+	+	+	+	+
Santoso et al. (2020)	+	?	−	−	+	−	+	+	+	+
Wang et al. (2020)	+	?	−	−	−	+	+	+	+	+
Crouzier et al. (2020)	+	−	−	+	+	+	+	+	+	+
Oatess et al. (2021)	+	?	−	+	+	+	+	+	+	+
Resasco et al. (2021)	+	?	+	+	+	+	+	+	+	+
Cabrera-Muñoz et al. (2022)	+	?	−	+	+	+	+	+	+	+
Ramírez-Rodríguez et al. (2022)	+	−	+	+	+	+	+	+	+	+
Funabashi et al. (2023)	+	?	−	+	+	+	+	+	+	+

Before delving into the discussion of spatial complexity’s meaning based on the included studies (*n* = 32), the studies are first categorized as short-term (*n* = 5) and long-term (*n* = 27) enrichment interventions, based on whether the studies investigate the effect of interventions with durations shorter or equal to 15 days (short-term interventions) or only of interventions lasting more than 15 days.

Over 5 short-term studies, 3 employ the in-space spatial complexity change intervention (i.e., change of single elements inside the environment), while the remaining studies utilize the Hamlet complex maze (*n* = 2). In particular, Leal-Galicia et al. (2007) conducted an intervention for 15 days, Birch et al. (2013) assessed the minimal effective duration by conducting an EE intervention for 2, 3, or 6 weeks, while Leger et al. (2015) conducted an intervention for 24 h, 1 week, 3 weeks, or 5 weeks. Some studies aimed to explore the minimal effective duration, considering the interplay of using running wheels and changes in spatial complexity across the three durations. On the other hand, Crouzier et al. (2018) and Crouzier et al. (2020) conducted a spatial complexity intervention for 2 weeks with a constant increase of layout complexity (the Hamlet complex maze), but no change of the in-space spatial complexity. The Hamlet test is a novel complex maze designed to assess behavioral responses and brain plasticity in mice. It consists of a circular apparatus with a central agora and five arms leading to functionalized houses with various stimuli. Mice are trained in groups to develop route learning strategies in this complex environment. The maze is equipped with compartments associated with specific tasks such as eating, drinking, hiding, running, or interacting with other mice. Training periods can last from 1 to 4 weeks, during which mice are placed in the maze for 4 h daily. A detailed summary of short-term interventions (*n* = 5) is presented in Table 3, showing duration, frequency of complexity change, and duration-dependent neurogenesis.

**Table 3:** Short-term spatial complexity duration effectiveness to stimulate neurogenesis.

Study	Short-term neurogenesis stimulation effectiveness							Running wheels	Frequency of changing complexity
	24 h	1 week	7–10 days	14–15 days	3 weeks	5 weeks	6 weeks		
Leal-Galicia et al. (2007)				+				yes	daily
Birch et al. (2013)				–	–		+	no	once/week
Leger et al. (2015)^†^	–	–			+	+		yes	twice/week
Crouzier et al. (2018)^*^				+				no	N/A
Crouzier et al. (2020)^*^				+				no	N/A

†Neurogenesis was observed from week 3, but neuronal survival at 5 weeks.

*Use of a complex layout without changing in-space or navigational complexity.

Over 27 long-term studies, 19 employ a consistent in-space spatial complexity change intervention (i.e., change of single elements inside the environment), while the remaining studies employ: gradual cage size increase followed by constant in-space complexity (*n* = 1); increase, decrease and constant complexity combinations (*n* = 1); the Marlau cage (*n* = 2); various intermittent complexity interventions (*n* = 4) in which the housing condition itself is changed across time and exposure to complexity may vary from low to high or vice versa depending on the phase of the intervention. Table 4 lists a summary of the frequency of complexity change, intervention duration, the provision or exclusion of running wheels to stimulate physical activity, and, last but not least, the age of the mice or rats.

**Table 4:** Long-term spatial complexity duration effectiveness to stimulate neurogenesis.

Study	Long-term neurogenesis stimulation effectiveness (months)^†^																				Running wheels	Frequency of changing complexity^††^	Starting age (rodent)
	1	2	3	4	5	6	7	8	9	10	11	12	13	14	15	16	17	18	19	20			
Kempermann et al. (1997)	+																				Yes	?	3 weeks
Kempermann et al. (1998a)	+	+																			Yes	?	6 and 18 months
Kempermann et al. (1998b)	+																				Yes	?	3 weeks
Nilsson et al. (1999)	+	+																			No	Every 3–4 days	3 months
Åberg et al. (2005)		+																			Yes/no	Intermittent	5 weeks
Segovia et al. (2006)		+																			Yes	Every 3–4 days	2 and 25 months
Hattori et al. (2007)	+																				Yes	Once / week	10 weeks
Leal-Galicia et al. (2008)																		+			Yes	Intermittent	3 months
Freret et al. (2012)						+															Yes	Twice / week	3 and 14 month
Fares et al. (2013)^*^			+																		No	3 times / week	3 weeks
Speisman et al. (2013)		+																			No	Intermittent	5–8 and 20–22 months
Grégoire et al. (2014)	–																				Yes/no	Alternating	2 months
Ramírez-Rodríguez et al. (2014)	+																				Yes	Every 3rd day	6 months old
Hosseiny et al. (2015)	+	+																			Yes	Twice / week	4 or 8 weeks‡
Hüttenrauch et al. (2016)											+										Yes	Weekly	1 month
Neidl et al. (2016)						+															No	5 times / week	18 months old
Zhang et al. (2016)	+																				Yes	Once, twice / week	adult
Birch and Kelly (2019)																				+	No	Each week	cohort
Sadegzadeh et al. (2020)	+																				Yes	Once / week	3 weeks
Sakhaie et al. (2020)	+																				Yes	Once / week	3 weeks
Santoso et al. (2020)^*^		+																			Yes	3 times / week	7 months
Wang et al. (2020)		+																			Yes	Twice / week	4 weeks
Oatess et al. (2021)			–																		No	Every 2 weeks	4 weeks
Resasco et al. (2021)		+																			No	Incremental	3 to 4 weeks
Cabrera-Muñoz et al. (2022)	+																				Yes	Every third day	3 weeks
Ramírez-Rodríguez et al. (2022)	+																				Yes	Varies^**^	10 weeks
Funabashi et al. (2023)	+																				No	Varies^***^	11 weeks

†Intervention durations are approximated to the month unit.

††When a change of environment is mentioned but the frequency is not specified, ‘?’ is reported in the table.

‡EE enhanced neurogenesis in juvenile but not young adult mice.

*Change of navigational, not in-space, complexity.

**Only for groups exposed to gradual increase of complexity, every third day one or two toys or tunnels were added to the environment till the third week. After the third week, only for the group with continued complexity, the location of the objects was changed twice a week.

***Cage enlargement took place after every 5 days, while the objects, which were present only in the last phase of the intervention (3 weeks of exposure to the most complex cage), were changed twice a week.

Firstly, out of all long-term in-space spatial complexity change interventions (*n* = 19), most studies (*n* = 14) combined the inclusion of running wheels with a change of in-cage complexity from once to five times per week, showing the effectiveness of a 1 to 3-month intervention in stimulating neurogenesis (Kempermann et al., 1997, 1998a, 1998b; Nilsson et al., 1999; Segovia et al., 2006; Hattori et al., 2007; Ramírez-Rodríguez et al., 2014; Hosseiny et al., 2015; Zhang et al., 2016; Sadegzadeh et al., 2020; Sakhaie et al., 2020; Wang et al., 2020; Resasco et al., 2021; Cabrera-Muñoz et al., 2022). Regarding the remaining long-term in-space spatial complexity interventions, one study by Oatess et al. (2021) investigated the effect of a single structural enrichment element (an acrylic tunnel), finding it was insufficient to affect hippocampal neurogenesis or markers of stress, two studies proved that a 6-month-long intervention for older subjects was effective with and without running wheels (Freret et al., 2012; Neidl et al., 2016), a cohort study lasting 20 months demonstrated the effectiveness of the intervention in stimulating neurogenesis without running wheels and with the least frequency of changing in-cage complexity (Birch and Kelly, 2019), and a 11-month-long intervention with combined running wheels and weekly changes in in-cage spatial complexity for 1-month-old subjects was effective in stimulating neurogenesis (Hüttenrauch et al., 2016).

Secondly, Ramírez-Rodríguez et al. (2022) explored the effects of different EE protocols on doublecortin cell maturation in the granular cell layer of the hippocampus and anxiety levels in mice. The mice were divided into five groups, each experiencing a unique housing condition: (1) standard conditions without EE, (2) constant EE complexity, (3) gradual increase of EE complexity followed by a gradual decrease, (4) gradual increase of EE complexity followed by constant EE, and (5) constant EE followed by a gradual decrease. The results showed that voluntary physical activity, either alone or in the context of a complex environment, increased doublecortin cell maturation in the granular cell layer of the hippocampus. This finding suggests that both physical activity and environmental complexity play a role in promoting the maturation of newly generated neurons in the hippocampus. Furthermore, the study found that any change in environmental complexity over time, regardless of the specific pattern, led to a reduction in anxiety levels in the mice. This indicates that dynamic alterations in the living environment may have a positive impact on emotional well-being, potentially through mechanisms related to novelty and adaptability.

Thirdly, Funabashi et al. (2023) investigated the effects of gradually enriching the housing environment on hippocampal neurogenesis in mice. The enrichment process involved progressively increasing the size of the housing space and introducing objects. The mice were initially housed in a standard cage for 5 days, then transferred to a large cage for 5 days, followed by an extra-large cage for 5 days, and finally, an extra-large two-floors cage with various objects for the remaining 21 days. The study tested the effect of the entire intervention at the end while physical activity was measured during each phase.

Fourthly, Fares et al. (2013) and Santoso et al. (2020) have used the Marlau cage. The Marlau cage is a specialized environment designed to provide continual cognitive stimulation for rats. It consists of a ground floor with two compartments: one containing food pellets and the other with water bottles. The upper floor houses a maze, and to access food, rats must climb from the lower compartment through the maze and down a slide tunnel. Access to water is provided via a one-way door. The maze configuration is changed regularly to maintain novelty and cognitive stimulation, with six different mazes offering a total of 12 configurations. Additionally, rats can enter and exit the maze through gates on each side to avoid territorial dominance. The cage provides a larger exploration area than conventional cages, encouraging physical activity. Rats housed in Marlau cages demonstrated enhanced brain plasticity, as evidenced by increased cortical thickness, hippocampal neurogenesis, and elevated levels of genes involved in tissue remodeling. Moreover, these rats exhibited improved spatial learning and memory, reduced anxiety-related behaviors, and better recovery of basal plasma corticosterone levels following acute stress (Fares et al., 2013). In comparison to the standard housing condition, the Marlau cage successfully increased the hippocampal BDNF and NGF levels when combined with physical exercise (Santoso et al., 2020). Interestingly, even compared to physical exercise alone, the combination of Marlau cage plus physical exercise led to enhanced BDNF levels, suggesting an additive effect for EE and wheel running (Santoso et al., 2020).

Lastly, regarding intermittent spatial complexity interventions, multiple studies used different techniques. Åberg et al. (2005) implemented a 2-month intervention using running wheels, with groups assigned to standard housing, enhanced housing, or alternating between the two groups with and without running wheels to stimulate physical activity. It was discovered that intermittent individual housing every second day without running wheels increased the survival of proliferated cells, suggesting a successful strategy for promoting survival of newly stimulated neurons without relying solely on physical activity. Leal-Galicia et al. (2008) conducted an 18-month intervention using running wheels and intermittent exposure to complexity for 3 h once per week to prevent habituation, resulting in an increased number of newly generated neurons, although no information was provided regarding cell survival. Speisman et al. (2013) implemented a 10-week intervention providing daily access to an EE for 2–3 h, with objects changed daily to maintain novelty, resulting in enhanced survival of new cells. Grégoire et al. (2014) utilized a 1-month alternating EE paradigm, revealing that while running significantly stimulated adult hippocampal neurogenesis, alternating EE did not substantially influence the neurogenic process, unlike other studies on changing spatial complexity. In conclusion, intermittent spatial complexity represents an intriguing area of research that warrants further exploration to draw definitive conclusions.

Before delving into the translatability of results and comparing the complexity of human versus rodent living conditions, it is important to revisit the earlier study by Clemenson et al. (2015), which demonstrated similar neurogenesis, spatial pattern separation, and place cells between rodents and humans. It is crucial to explore any differences between the diverse rodent species and strains used in the studies included in this systematic review. Amrein et al. (2011) provided a quantitative comparison derived from seven laboratory strains of mice (Balb, C57BL/6, CD1, outbred) and rats (F344, Sprague–Dawley, Wistar), along with six other wild-derived rodent species, concluding that absolute age is the critical factor regulating cell genesis in the adult hippocampus. The strains mentioned in the study by Amrein et al. (2011) are also included in the studies analyzed in this systematic review. Specifically, the included mouse strains are Balb, C57BL/6, and CD1, while the rat strains include F344, Sprague–Dawley, and Wistar. However, additional strains are included in the systematic review but were not mentioned in the study by Amrein et al. (2011), such as NMRI mice, Swiss mice, FVB/N mice, 129/SvJ mice, and Evans rats.

To address this gap, it’s worth noting that FVB/N mice were found to exhibit learning abilities comparable to the C57BL/6 strain in a non-visual hippocampus-dependent task. Mansk et al. (2023) provided multiple evidence supporting that EE affects social memory and adult hippocampal neurogenesis independently of mouse strain. Additionally, Kempermann et al. (1998b) highlighted that 129/SvJ mice do not perform well in learning tasks, yet EE was a successful intervention regardless of strain differences. Although no studies directly compare NMRI mice and Evans rats strains, they are both used in a small subset of the included studies (*n* = 3), which may not significantly impact translatability but may warrant further investigation.

## 4 Discussion

Overall, (*n* = 32) studies support the argument that spatial complexity stimulates neurogenesis and correlates positively with hippocampal plasticity outcomes. As shown in Tables 3, 4, the majority of studies focus on changing in-space complexity by interchanging or replacing elements, even in short-term studies. It is noteworthy that the presence of running wheels and the frequency of changing spatial complexity per week appear to have additive effects on neurogenesis. Indeed, the combination of running wheels and high number of changes of spatial complexity appears to reduce the duration of the enrichment intervention required to obtain increased neurogenesis. For instance, Grégoire et al. (2014) found that during a 4-week period of housing the mice, running significantly stimulated adult hippocampal neurogenesis, while environmental complexity, including novelty, did not exert a substantial influence on the neurogenic process. However, Birch et al. (2013) found that 6 weeks of structural EE alone, with weekly changes of EE complexity, were sufficient to increase the nerve growth factor (NGF) concentration, subgranular progenitor cell survival in the dentate gyrus, and synaptogenesis, all without the presence of running wheels. Importantly, in these two studies mice were adults of comparable age (2 and 3 months old at baseline, respectively), which eliminates age from acting as an interfering variable. The study by Birch et al. (2013) also supports the findings reported by Grégoire et al. (2014) because the former indicated that without physical activity, durations of 2 and 3 weeks were not sufficient to induce the neurogenic effect, contrarily to the 6-weeks duration. To further support this argument, Leal-Galicia et al. (2007) found that 2 weeks were sufficient to show a positive effect on neurogenesis but only when running wheels were used along with a daily change of spatial complexity. If we compare the results of the latter study with the findings reported by Leger et al. (2015), who found that a twice-per-week change of complexity combined with running wheels induced neurogenesis under both 3- and 5-week durations, but not 1-day or 1-week durations, we can conclude that the presence of running wheels and a high frequency of change of spatial complexity have additive effects, and that their combination can shorten the intervention duration necessary for an effective stimulation of neurogenesis.

However, the intervention of spatial complexity consistently succeeded in stimulating neurogenesis, manifested as increased BrdU count, and BDNF levels. Due to the complexity of neurogenesis and the presence of multiple complexity models, this systematic review was unable to perform meta-analysis. Instead, it attempted to define spatial complexity and identify the factors potentially modulating its effects on neurogenesis. This systematic review culminates in proposing a quantitative regression model, which may be useful both for synthetic description of rodent EE models and for translation of results from rodents to humans.

Based on Tables 3, 4, this systematic review proposes a regression model involving the duration of spatial complexity intervention, physical activity, frequency of changing in-space spatial complexity, and age. While the effect of housing space volume has been explicitly investigated only in one study that gradually increased the cage size but did not effectively explore the impact of this intervention before adding element complexity (Funabashi et al., 2023), we consider housing space volume as another prospective independent variable for more precision, knowing that the rest of studies used larger cages for any EE intervention compared to any standard housing condition. However, we confidently exclude the social variable because some studies support that neurogenesis is present in both social isolation + EE (spatial complexity) and group housing + EE (spatial complexity), resulting in neurogenesis compared to both groups without EE (Madronal et al., 2010; Monteiro et al., 2014).

While not addressed in this study due to its focus on complexity, it is crucial to acknowledge that temperature plays a significant role similar to physical activity in stimulating neurogenesis. Therefore, we purposefully include it as another independent variable because in the case of humans, temperature and physical activity can interact simultaneously. BDNF levels are known to fluctuate depending on heat exposure. Passive heat exposure has been shown to modulate BDNF, as indicated by a study by Kojima et al. (2018), where serum BDNF levels increased by 66% following a 20-min immersion in 42°C water, remaining significantly elevated for 15 min post-immersion. Additionally, a study by Koyama et al. (2018) demonstrated that short-term heat exposure promoted hippocampal neurogenesis in rats when exposed daily to a 1-h heat treatment at 36°C over a 7-day period, likely through enhanced levels of vascular endothelial growth factor (VEGF), a molecule belonging, like BDNF, to the class of growth factors.

Long-term factors such as sunlight exposure, time of year, and seasonal variation have also been reported to influence BDNF levels (Molendijk et al., 2012). This study found variations in serum BDNF concentrations across seasons, with higher concentrations observed in the spring–summer period and lower concentrations in the autumn-winter period. Additionally, the number of sunshine hours positively correlated with serum BDNF concentrations. In summary, there is a clear relationship between whole-body energy status, adult hippocampal neurogenesis, and neuron survival (Landry and Huang, 2021).

Last but not least, it is critical to distinguish between the frequency of changing spatial complexity (in-space or layout) and the diversity of complexity (i.e., the diversity among the enrichment items provided within a same period). This is implicitly derived from looking at a number of studies. The only two studies that highly increased space-use diversity through the layout prior to the beginning of the experiment without having to provide running wheels or change spatial complexity later on showed that this intervention was sufficient to increase hippocampal neurogenesis, in comparison to standard housing which featured a poorer variety of environmental items (Crouzier et al., 2018, 2020). Hence, it appears that the diversity, variety or degree of complexity is a distinct factor that is sufficient to induce neurogenesis independently from the change of spatial complexity. For instance, the study by Oatess et al. (2021) has not used running wheels nor a complex EE, but just one single structural element of enrichment (an acrylic tunnel), and no hippocampal neurogenesis was observed. Reliance on a single structural enrichment only may have reduced the experienced diversity of complexity, which could explain the lack of hippocampal neurogenesis. Cages and tunnels were replaced with identical cages and tunnels every 3 weeks, which may have further limited neurogenesis stimulation. Furthermore, all other included studies employed EE interventions that featured structural elements with different shapes, functions and colors, further highlighting the importance of the diversity of the available enrichment items, in addition to change of enrichment items across time. Last but not least, we have evidence from a study conducted on humans that higher diversity of activities leads to greater hippocampal volumes across both hemispheres (Urban-Wojcik et al., 2022).

Thus, we propose the long-form definition of spatial complexity to be understood through the following regression equation (Equation 1):


(1) $$N=\beta_{0}+\beta_{1}D+\beta_{2}P+\beta_{3}L+\beta_{4}V+\beta_{5}F+\beta_{6}S+\beta_{7}A+\beta_{8}T+\epsilon$$

where:

N: Neurogenesis.

*D*: Duration of intervention.

*P:* Physical activity.

*L:* Spatial layout complexity: number of distinct spaces.

*V:* Diversity of complexity items’ characteristics.

*F:* Frequency of changing spatial complexity: navigational and/or in-space.

*S:* Size of the living space: Volume and/or area.

*A:* Age of the subject.

*T:* Temperature.

β _0_, β _1_, β _2_, β _3_, β _4_, β _5_, β _6_, β _7_, β _8_: Regression coefficients representing the relationship between each independent variable and the dependent variable (neurogenesis).

ϵ : Error term.

We anticipate that neurogenesis itself may be further broken down into detailed regression models. While the majority of existing publications supports the stimulation of neurogenesis in the form of producing new cells, specific studies have indicated that certain independent variables affect cell survival, synaptogenesis, and plasticity in CA1 and CA3 areas. Therefore, Equation 1 refers to the general role of the adult hippocampus in producing new cells, but subsequent processes are more intricate and require further investigation. Different regression models should be separately tested for specific processes and for rodents and humans independently to yield more accurate results.

However, our aim is to explain each of the independent variables separately and address the diversity of rodent species before proceeding with the translatability and testability of models for human subjects. Gender and age are two important independent variables sensitive to spatial complexity intervention differently. Several studies have used females instead of male rodents to mitigate male agonistic behaviors in response to changes in complexity and other factors. Whether this sensitivity exists in humans remains unknown. Regarding age, the included studies reported that spatial complexity successfully stimulates neurogenesis across all rodent age groups, even when the reported outcomes and duration varied.

In order to understand how to translate the duration of the enrichment intervention from rodents to humans, we should first consider the rodent-human translation of age. For example, rats undergo rapid development during childhood and reach sexual maturity by the sixth week of age. By adulthood, each month of a rodent’s life is approximately equivalent to 2.5 human years (Andreollo et al., 2012). Another comprehensive review (Dutta and Sengupta, 2016) indicates that mice reach sexual maturity averagely at 10 weeks of age, while humans reach it at 20 years. Hence, at adulthood onset, for the past lifetime of mice, 2.6 mouse days are equivalent to 1 human year. A mouse at the weaning period of 28 days is equivalent to a 6-month-old infant, while a 15-month mouse (the age of reproductive senescence) is equivalent to a 51-year-old human, and a mouse aged 24 months is equivalent to a 80-year-old human. According to the criteria presented by Dutta and Sengupta, in the period between adulthood onset and reproductive senescence onset (from 10 weeks to 15 months in mice, and from 20 years to 51 years in humans), 1 mouse day corresponds to 4.2 human days.

Hence, the translatability of any of the interventions should be sensitive to the reported rodent age, depending on the intended human age group—whether infant, adult, or elder—where EE is questioned for application (Baroncelli et al., 2010; Morgan et al., 2013; McDonald et al., 2018; Flores-Ramos et al., 2022; Rojas-Carvajal et al., 2022).

Therefore, we cannot expect a 1-month spatial complexity intervention conducted on an adult rodent to be equivalent to a 1-month intervention on a human subject. In fact, it may be equivalent to more than 4 months for an adult human subject. This underscores another critical point to consider—the frequency of changing in-space physical complexity, which can be as simple as altering sensory elements. Such changes have been found to be effective alone, without the use of running wheels (Birch and Kelly, 2019), yet in humans they may require longer durations, hypothetically.

Running wheels emerge as a constant element across most of the included studies. In the case of humans, instruments for running generally are not present in the house environment, apart from the minority of people keeping fitness treadmills. Hence, an effective spatial enrichment in humans may hypothetically require more frequent changes in in-space complexity. In that regard, spatial complexity can become an interesting area for welfare promoting architectural experimentation, especially given that the architectural experience through spatial complexity is already an emerging area in neurodesign and architectural research. This area entangles aesthetic responses to architectural experiences across three scales: fascination, coherence, and hominess, along with the complexity of the layout itself and its unusualness (Coburn et al., 2020; Gregorians et al., 2022).

Space syntax or GPS tracking can aid in exploring walking patterns and quantifying physical activity in the form of walking (Sharmin and Kamruzzaman, 2018; Zaleckis et al., 2022). Whether at the urban or house scales, spatial configuration and walkability can be experimented with using GPS tracking and space syntax.

Translation of rodent results to humans can be achieved at the micro scale, such as the house or architectural space, through changing single interior elements of the in-space complexity, or it can be obtained by changing the whole spatial complexity layout itself. The effectiveness of employing a layout with high spatial complexity is supported by the two studies using the Hamlet complex maze (Crouzier et al., 2018, 2020), representing a micro scale, and the two studies using the Marlau cage (Fares et al., 2013; Santoso et al., 2020), representing the macro scale. In terms of translatability, the Hamlet complex maze resembles a human courtyard-style or apartment residential typology, while a standard or enriched cage can be a model of a human single-room apartment or studio. Some studies utilized a two-story cage design at some points in the intervention, giving a spectrum of typologies to explore using human subjects in their daily habitual residences. Mice in the Hamlet complex maze were in their adulthood, supporting the stimulation of adult hippocampal neurogenesis (AHN) throughout life using that typology. While the Hamlet maze is complex, consisting of distinct room-like spaces (eating, drinking, running, social interaction and hiding areas) with an agora in the center, but constant in terms of no change taking place, the Marlau cage is designed to provide an extremely complex maze between two simply enriched spaces, with a maze that is changed three times a week. Both the Hamlet constant-complexity maze and the Marlau cage with changing navigational maze designs appear to be directly translatable spatial complexity models that can help assess the outcomes of house typologies, such as single apartments or urban residential environments, respectively.

In the case of human subjects, one possible model to dissect the effects of spatial complexity is the one where the human house (H) is considered analogous to the rodent impoverished cage, and is compared to the house and the city combined (H + C) as an analog of a more complex rodent cage. Support to this model is provided by a study recently done by McCormick et al. (2022) who found, using GPS signals, that homebodies demonstrated significantly poorer cognitive function than venturers, but some limitations were the small sample size (*n* = 117) and the fact that the participants were recruited from community mental health centers and reported to have serious mental illness. Because the lifestyle of humans is variable, the equation can be made more complex as follows (H + C+…n) because in a single day, the workplace (W), visits to other houses (V), shopping malls (S), and several spaces external to the house may be part of the spatial complexity experienced during a day. The house just takes priority because it generally remains constant for years or decades, mostly unchanged, and because people spend a great amount of their lifetime at home. Hence, while focusing on the house and city combined (H + C), it is important to highlight the variety of house typologies and the various complexities of different cities. The latter may be easily tracked by quantifying the spatial complexity at the macro level and using GPS to track the daily lifestyle as it was done by Shin et al. (2024). There might be differences between individuals of a same city at the level of lifestyle, while there might be differences between cities regarding the structural complexity independently from the lifestyle of their inhabitants.

While research on intermittent complexity is still immature, we can observe an interesting translation to humans during the COVID-19 pandemic lockdown, quarantine, stay-at-home, or shelter-in-place interventions through comparisons between decreased and increased complexity. To elaborate, those restrictions led to a decrease in spatial complexity followed by an increase in spatial complexity. Because it was difficult to explore the impact of such lockdown or quarantine on neurogenesis *per se*, indicators of anxiety can be alternative outcomes to assess the effects of spatial complexity, knowing that an intervention reported recently on rodents by Ramírez-Rodríguez et al. (2022) found that any change in complexity over time reduced anxiety. Thus, if we translate, then it would be (H + C) followed by (H-C) as the first wave of lockdown, then followed by (H + C), then followed by (H-C), and finally (H + C). We can support this assumption with findings from Italy and the United Kingdom through the UK Household Longitudinal Study (UKHLS) conducted on large samples and at different points in time before, during, and after the waves of lockdown (Niedzwiedz et al., 2021). Di Blasi et al. (2021) investigated the change in psychological distress in 1,129 subjects during and post COVID-19-related lockdown, finding that depression, anxiety, and stress formed a spatially contiguous pattern that remained unchanged in both waves. Similar findings were reported in another study in Italy (Benfante et al., 2022). A study using the UKHLS database on (*n* = 9,748) concluded that psychological stress increased 1 month into the initial phase of lockdown, which we interpret as a decrease in complexity, yet we cannot rely on the isolation period alone without considering the subsequent increase in complexity. Another study by Daly and Robinson (2022) using the UKHLS database on a large sample (*n* = 10,657) after the second COVID-19 wave revealed that clinically significant distress rose during the second wave, with similar levels to those in the aftermath of the first wave. Before we reject or accept the null hypothesis that intermittent complexity reduces anxiety and depression, which may be considered psychological indicators of neurogenesis, we must mention that anxiety and depression reported during COVID-19 may have been influenced by both the change in spatial complexity and the fear of the pandemic itself, including the fear of contagion and of death. Above all, we must measure anxiety and depression symptoms at different points before and after the COVID-19 pandemic, during each wave, between the two waves, and after the pandemic is over to confidently support whether intermittent complexity was effective on humans or not. This is another area of research that can be conducted using existing UKHLS datasets at different points in time.

For the future, an interesting line of inquiry would be the investigation of the causal relationships between EE, neurogenesis and cognitive-enhancement. An approach that can be particularly useful to understand such links is the adoption of experimental paradigms in which EE is provided to mice with suppressed neurogenesis. For instance, Bogado Lopes and colleagues employed cyclin D2 knockout mice, which constitutively have extremely low levels of adult hippocampal neurogenesis, and housed them for 3 months in a highly-complex enriched environment consisting of 70 connected cages, finding that in D2 knockout mice there is a reduction of the EE-induced enhancement of spatial cognition (Bogado Lopes et al., 2023). On the other hand, when mice that had underwent hippocampal X-Ray irradiation, which annihilates hippocampal neurogenesis, were exposed to EE, it was seen that hippocampal neurogenesis was not required for the EE-induced cognitive enhancement, suggesting that there may be independent mechanisms mediating the effects of EE on cognition, for example the upregulation of growth factors such as BDNF (Meshi et al., 2006). Future research is needed to fully understand the causal links between EE and enhanced cognition.

Furthermore, new paradigms could be employed in rodents to explore the effects of spatial complexity, namely: (a) smart cages, i.e., complex cages containing technological interactive elements that allow automated home-cage behavioral monitoring (Mingrone et al., 2020; Voikar and Gaburro, 2020; d'Isa and Gerlai, 2023; Kahnau et al., 2023; Lipp et al., 2024); (b) seminatural environments, i e. housing environments that reproduce in the laboratory elements of the natural habitat of the hosted species (Hernández-Arteaga and Ågmo, 2023); (c) freely accessible complex mazes connected directly to the home-cage, such as the automated eight-arm radial maze designed by the Julius Emmrich laboratory (Mei et al., 2020; Kohler et al., 2022); (d) radio-collars allowing radio-tracking of free-ranging rodents living in their natural habitat (Stradiotto et al., 2009; Bonacchi et al., 2021).

Indeed, in addition to the Hamlet maze and the Marlau cage, in the last decade many groups have developed systems that could be employed to investigate spatial complexity, for instance Eco-HAB, a large and ecologically-relevant four-compartment apparatus with covered areas and narrow tunnels which resemble rodent burrows (Puścian et al., 2016; Winiarski et al., 2022) or ColonyRack, in which mice can freely explore a very large and complex environment comprising 70 cages connected both horizontally and vertically (Zocher et al., 2020).

## 5 Conclusion

In conclusion, this systematic review confirms the positive impact of spatial complexity on neurogenesis and hippocampal plasticity. Through the analysis of 32 studies, we established a regression equation outlining key factors influencing neurogenesis. The importance of spatial complexity for neurogenesis may challenge the conventional interior design of human houses. Additionally, models like the Hamlet complex maze and Marlau cage may offer insights into how architectural design and navigational complexity influence neurogenesis. These findings underscore the potential of spatial complexity interventions to enhance cognitive health, informing future research and design strategies aimed at optimizing well-being. Translatability from mice to humans can be achieved by equating age and duration between rodents and humans. Moreover, data from the pandemic period may provide direct insights on the effects of changing spatial complexity in humans. This comprehensive review illuminates the intricate dynamic interplay between spatial complexity and neurogenesis, paving the way for innovative approaches to architectural and interior design that prioritize cognitive well-being, as well as urban-scale interventions. Those findings, along with the newly proposed regression model, can be translated into interventions on human subjects at different life stages, from early childhood to adulthood, to stimulate AHN. In the case of aging, they can be utilized to achieve cognitive reserve or reverse cognitive decline.

## Data availability statement

The original contributions presented in the study are included in the article/supplementary material, further inquiries can be directed to the corresponding author/s.

## Author contributions

MK: Conceptualization, Data curation, Formal analysis, Funding acquisition, Investigation, Methodology, Project administration, Resources, Validation, Visualization, Writing – original draft, Writing – review & editing.
